# Does architectural lighting contribute to breast cancer?

**DOI:** 10.1186/1477-3163-5-20

**Published:** 2006-08-10

**Authors:** Mariana G Figueiro, Mark S Rea, John D Bullough

**Affiliations:** 1 Lighting Research Center, Rensselaer Polytechnic Institute, 21 Union Street Troy, NY 12180 USA

## Abstract

**Objectives:**

There is a growing interest in the role that light plays on nocturnal melatonin production and, perhaps thereby, the incidence of breast cancer in modern societies. The direct causal relationships in this logical chain have not, however, been fully established and the weakest link is an inability to quantitatively specify architectural lighting as a stimulus for the circadian system. The purpose of the present paper is to draw attention to this weakness.

**Data Sources and Extraction:**

We reviewed the literature on the relationship between melatonin, light at night, and cancer risk in humans and tumor growth in animals. More specifically, we focused on the impact of light on nocturnal melatonin suppression in humans and on the applicability of these data to women in real-life situations. Photometric measurement data from the lighted environment of women at work and at home is also reported.

**Data Synthesis:**

The literature review and measurement data demonstrate that more quantitative knowledge is needed about circadian light exposures actually experienced by women and girls in modern societies.

**Conclusion:**

Without such quantitative knowledge, limited insights can be gained about the causal relationship between melatonin and the etiology of breast cancer from epidemiological studies and from parametric studies using animal models.

## Background

The incidence of breast cancer has grown continuously since the turn of the twentieth century in industrialized societies [1,2]. In 1978, Cohen and colleagues [3] put forward the hypothesis that the pineal gland might play a role in breast cancer etiology. In 1987, Richard Stevens [4] put forward the "melatonin hypothesis" as a possible explanation for at least part of the growing incidence of breast cancer [4]. According to his hypothesis, the high incidence of breast cancer in industrialized society was, perhaps, caused by electricity in modern buildings through reductions in melatonin concentrations, a hormone produced at night and under conditions of darkness. He postulated that one of the electricity-induced reductions in melatonin was a result of electric light at night (LAN). It is important to note that light is formally defined in terms of a specific visual response in humans [5]. It is technically incorrect to use the term light or lighting when referring to other species, or in relation to nonvisual (e.g., circadian) responses in humans. The term optical radiation is most accurate to describe the portion of the electromagnetic spectrum spanning ultraviolet, visible, and infrared radiation. However, the terms light and lighting are widely used to describe optical radiation in the biological and medical research community, and these terms are used interchangeably, albeit technically incorrectly, throughout this paper.

The melatonin hypothesis stimulated various lines of research, from laboratory studies using animal models to epidemiological studies with humans. Suppression of melatonin was hypothesized to increase production of estrogens in the ovaries, which in turn would stimulate the turnover of breast epithelial stem cells, thereby increasing the likelihood of cancer [3]. Low melatonin levels in rats were also shown to increase cancer cell proliferation in existing tumors [6,7]. Epidemiological studies showed night-shift work, a surrogate for LAN, increased the likelihood of breast and colorectal cancer [8-11]. Thus, although no direct causal link has ever been established between breast cancer and LAN in architectural spaces, several researchers have suggested plausible links between increased cancer risk and electric LAN through melatonin depletion or disruption.

It is widely accepted that light exerts a powerful influence on the human circadian system, including melatonin synthesis, and it is becoming more widely accepted that the circadian system plays a role in breast cancer [12]. For example, the efficacy of breast cancer treatment varies with circadian timing [13,14]. The present discussion focuses on light as it affects melatonin, a potential mediator of cancer development and growth.

To establish causal links between architectural LAN, melatonin levels and the growing incidence of breast cancer in the general population, it is first and foremost necessary to accurately specify the light stimulus (in terms of quantity, spectrum, distribution, duration and timing [15]) as it affects the human circadian system in industrialized societies. Without this critical first step it is very difficult to ascribe increased risk of breast cancer in women living in industrialized societies to architectural lighting. The purpose of this paper is to focus attention on the development of a more quantitative characterization of *circadian light *experienced by women and girls in modern societies and, in particular, to begin to bridge that understanding to epidemiological studies as well as to parametric laboratory studies using animal models.

The hormone melatonin is synthesized by the pineal gland at night and under conditions of darkness in mammals [16]. Synthesis and release of melatonin follow a robust circadian rhythm and are highly governed by the light-dark cycle. Reiter and colleagues [17] have shown that melatonin participates in various physiological processes, including immune system functions. Melatonin can prevent damage to DNA, and DNA that is not repaired can mutate and initiate cancer [18,19]. Melatonin also participates in the regulation of circadian rhythms of cell metabolism and inhibition of chemically-induced carcinogenesis in rats as well as the growth of tumors in rats [6,7,20].

Very recently, Blask and colleagues [20] showed that exposure to different levels of white fluorescent light during the 12-hour dark phase resulted in a dose-dependent suppression of melatonin in rats bearing rat hepatomas or human breast cancer xenografts. Further, they showed that exposure to increasing levels of white light (from 0 to 345 μW/cm^2^) resulted in dose-dependent stimulation of tumor growth and linoleic acid uptake and metabolism. Relatively low light levels (0.1 μW/cm^2^) suppressed nocturnal melatonin in these rats. Blask and colleagues also showed that human nocturnal melatonin signals inhibit activities such as linoleic acid uptake that are associated with human breast cancer growth and that this effect is diminished by ocular exposure to bright, white light at night. They exposed women to 2800 lux of white light (580 μW/cm^2^) at eye level, which resulted in an approximately 40% reduction of melatonin levels in blood compared to darkness levels. Perfusing tumors bearing human breast cancer xenografts with this melatonin-depleted human blood resulted in increased linoleic acid uptake in the xenografts, which is related to increased cancer growth [20]. Although this landmark study is limited to an indirect relationship between melatonin and cancer growth rates, melatonin depletion might also have important implications for cancer development [21].

Also, consistent with Stevens's [4] melatonin hypothesis, epidemiological studies suggest that night-shift work is associated with an increase in breast and colorectal cancer risk, potentially mediated through melatonin suppression by exposure to LAN. Tynes and colleagues [8], Hansen [9] and Davis and colleagues [10] studied women performing night-shift work and all of the authors concluded that female night-shift workers were at higher risk of breast cancer compared to daytime workers. Two prospective cohort studies utilizing data from the Nurses' Health Study [22] showed a relative risk of 1.36 (95% CI, 1.04–1.78) associated with 30 years or more of rotating night-shift work and a relative risk of 1.79 (95% CI, 1.06–3.01) associated with 20 years or more of rotating night-shift work, after controlling for known breast-cancer risk factors [11].

Finally, epidemiological studies suggest that blind women, who are less likely to suppress melatonin by LAN, are at lower risk of breast cancer than sighted women [23]. Although these results also suggest that LAN may be linked to an increased risk of breast cancer, Lockley and colleagues [24] showed that the urinary metabolite of melatonin (aMT6s) output (mg of aMT6s per 24 h) and amplitude (micrograms per h) of blind women with and without light perception did not differ.

Recommendations for lighting practice are being made based on extrapolations from these animal and epidemiological studies [25]. However, without proper quantification of LAN as it affects the human circadian system outside laboratory conditions, particularly those using animal models, generalizations from research linking melatonin to cancer initiation and progression cannot be meaningfully made and implications for architectural lighting cannot be responsibly proposed. Again, this paper attempts to lay the foundation for a more complete, quantitative characterization of circadian light in the built environment as a fundamental step toward understanding the links between light, circadian regulation, melatonin and breast cancer risk.

### Light in Architectural Spaces

Although perhaps surprising to those outside the field of architectural lighting, incandescent residential lighting levels are typically lower than those experienced in offices and much lower than those experienced outdoors during the daytime. General ambient light levels on the floor or other horizontal surfaces in a home rarely exceed 300 lux [26]. Even for special visual tasks in residences, such as grooming and cooking, vertical light levels likely to be incident on the face or eyes are usually between 50 and 200 lux, and light levels on the visual task rarely exceed 500 lux. Light levels from fluorescent lamps on task areas in commercial and industrial spaces can be two to three times higher, and those outdoors from daylight and sunlight can be 20 to 100 times greater. In buildings illuminated by ceiling luminaries containing fluorescent lamps, which direct light downward, light levels at the cornea are about one-fifth of those measured on a horizontal surface, so in the home, ambient illuminances from incandescent lamps rarely exceed 200 lux at the cornea, and more typically at night are between 10 and 100 lux, based on measurements of vertical illuminances in several residences (Table 1[27]) and based on data obtained with a new instrument designed to record and store (for up to a week at a time) both conventional photopic illuminance and circadian light exposure measurements made at the plane of the eye [28].

**Table 1:** Vertical (at the Cornea) Photopic Illuminance Measurements [27] in Several Interior and Exterior Locations

**Location**	**Median (lx)**	**Minimum (lx)**	**Maximum (lx)**
windowed offices (78 locations in 12 rooms)	201	24	2050
windowless offices (68 locations in 12 rooms)	95	22	271
hospital patient rooms (53 locations in 5 rooms)	121	15	1182
hospital nurse stations (66 locations in 9 rooms)	126	22	837
hospital common areas (16 locations in 2 rooms)	319	26	1357
residential kitchens (48 locations in 5 rooms)	11	4	73
residential bedrooms (14 locations in 4 rooms)	43	8	170
parking lots (120 locations in 4 lots)	7	1	95

**Figure 1:**
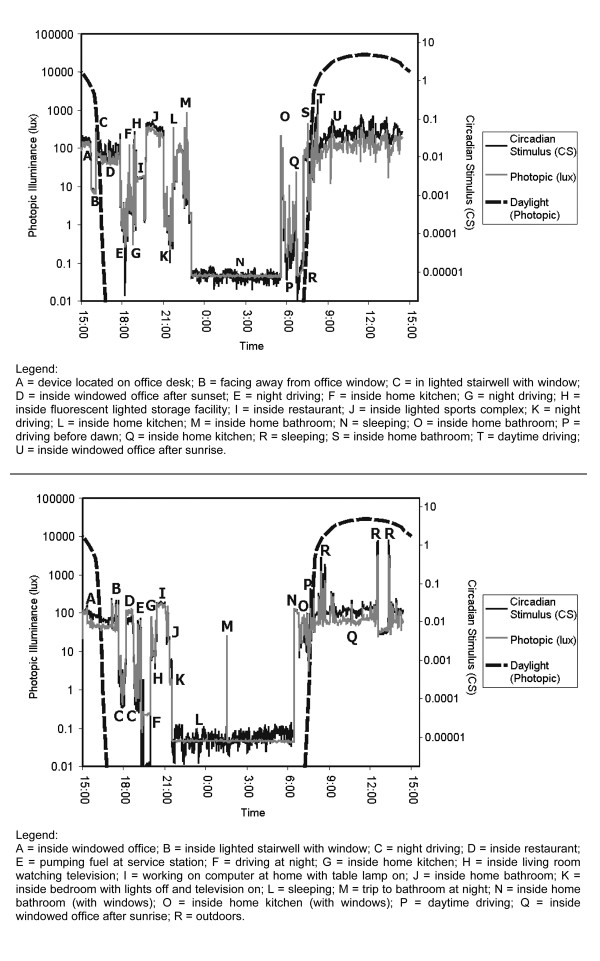
Light profiles, in photopic illuminance (solid gray, left ordinate scale) and in circadian stimulus (solid black, right ordinate scale) units [37], for two women working in daylighted offices in Troy, NY, USA. Measurements were made continuously starting at 15:00 on 1 December 2005 until 14:30 on 2 December 2005. Also shown are vertical photopic illuminances (dashed black, left ordinate scale) expected from outdoor exposure to a partly cloudy sky at the same location on the same date in open country for a 45° azimuth angle from the sun [26].

**Figure 2:**
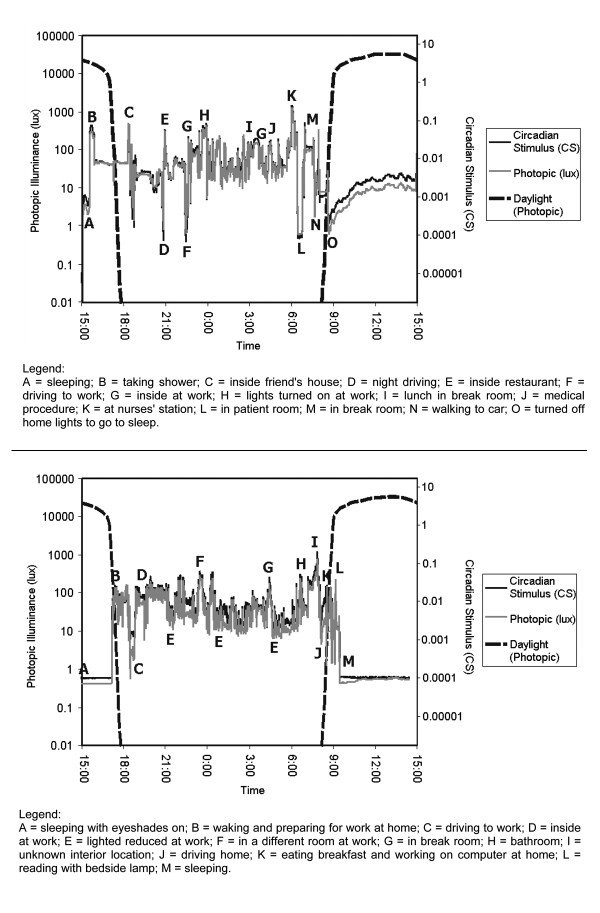
Light profiles, in photopic illuminance (solid gray, left ordinate scale) and in circadian stimulus (solid black, right ordinate scale) units [37], for two night shift nurses working in South Bend, IN, USA. Measurements were made continuously starting at 15:00 on 30 January 2006 until approximately 15:00 on 31 January 2006. Also shown are vertical photopic illuminances (dashed black, left ordinate scale) expected from outdoor exposure to a partly cloudy sky at the same location on the same date in open country for a 45° azimuth angle from the sun [26].

Figures 1 and 2 show representative profiles of circadian light exposure for two diurnal women working in a daylighted office during the day, and for two night-shift nurses, respectively, along with expected outdoor light exposures from daylight [26] at the same geographic location as the women represented in the two figures.

As seen in Figure 1, the two diurnal women working indoors were exposed to dim, extended and aperiodic circadian light exposure quite unlike a hypothetical woman working outdoors under a robust light-dark cycle, assumed to be ideal for regulating the circadian system. Note too that the times of light exposure for both of these day-shift women have been extended several hours beyond sunset and that the magnitude and the variation in these nocturnal light exposures are similar to those they experienced during the day. Interestingly as well, light exposure in public spaces from high intensity discharge or fluorescent lamps, such as in gymnasia, for example, are much higher than residential light exposures, and can be experienced for several hours, so these events, if experienced on a regular basis, could possibly be of greater impact on circadian regulation and melatonin production than the ambient lighting from incandescent lamps typically found in residences.

Figure 2 shows similar data to those in Figure 1 but for two female nurses who work during the night shift. These women also experience low light exposures relative to outdoor daytime light levels, but this would be expected because they are asleep during most of the daytime. These night-shift women also seem to have relatively lower light exposures during their waking hours than the day-shift women experienced during their waking hours.

It should be emphasized that the lighting profiles in Figures 1 and 2 are only "snapshots" of light exposure experienced by day- and night-shift workers. Much longer recording periods would likely be necessary to accurately predict circadian response to light because the sensitivity of the circadian system to LAN on a particular night will be affected by a person's light exposure history. Sensitivity to LAN in terms of melatonin suppression has been shown to be higher (i.e., less light is needed to suppress melatonin) after a prior history of exposure to low light levels and lower after a prior history of high light levels [29-31]. Thus, it probably will be necessary to track several days of light and dark exposures to begin to understand the impact of LAN on nocturnal melatonin suppression for people in their natural environments.

### Light in Animal Laboratories

To link architectural LAN to breast cancer risk, the lighting profiles experienced by people, such as those in Figures 1 and 2, ultimately need to be translated into parametric lighting studies with laboratory animals used as models for cancer research. Bullough and colleagues [32], after a literature review and calculations, showed that threshold levels of light (quantity, spectrum, distribution and duration) for activation of the circadian systems of nocturnal rodents commonly used as cancer models can be as much as 10,000 times lower than the threshold levels needed to activate the circadian systems of diurnal species such as humans. As suggested above, this profound species difference is clearly demonstrated in the work of Blask and his colleagues, where only 0.1 μW/cm^2 ^of white light was sufficient to reliably suppress nocturnal melatonin in rats [20,33], but 580 μW/cm^2 ^(2800 lux) was necessary to suppress nocturnal melatonin in women by 40% [20]. Not only is this level several orders of magnitude greater than the threshold for melatonin suppression in nocturnal rodents, it is equivalent to daytime light levels found outdoors during the morning and not at all similar to light levels produced by architectural LAN, especially in residences (Table 1). Further, experimental data suggest that nocturnal rodents such as mice [34] do not reliably exhibit spectral opponency in circadian phototransduction, although humans and diurnal primates do appear to exhibit opponency [35-38]. These comparisons among species underscore the importance of properly characterizing light as a stimulus to the circadian systems in both humans and in the nocturnal rodents used as animal models for understanding human breast cancer etiology.

Light levels in animal laboratories designed in the 1990s have typically been specified to be about 600 to 800 lux of white light on a horizontal plane one meter above the floor [39], corresponding to an irradiance of about 150 μW/cm^2^. More recently, recommendations [40] have been reduced to about 300 to 325 lux (about 70 μW/cm^2^) to help reduce the incidence of retinal damage in nocturnal rodents by lighting. Regardless, these light levels would still be likely to result in illuminances at the eyes of rodents housed in such laboratories well above the 0.1 μW/cm^2 ^threshold for melatonin suppression [20,33]. Such disparities between typical human light exposures and the conditions used in the study of cancer in nocturnal rodents will need to be much better addressed before a quantitative understanding of architectural LAN and breast cancer risk can be developed from laboratory studies where animal models are used.

### LAN as a Stimulus for Melatonin Suppression

**Figure 3:**
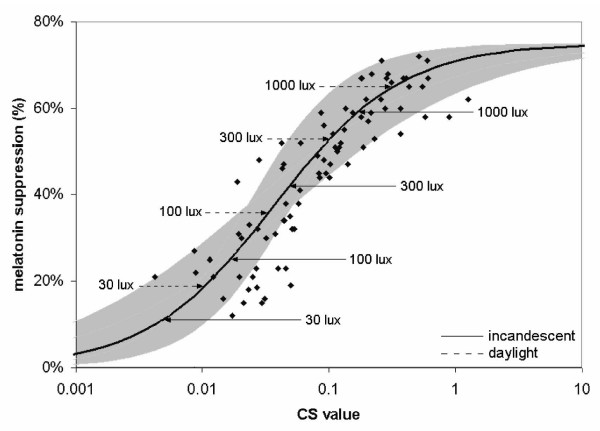
Melatonin suppression [35, 41-45] as a function of circadian stimulus (CS) [37]. Horizontal arrows show the corresponding CS value for varying corneal illuminances from incandescent (solid arrows) and daylight (dashed arrows) illumination [46]for one hour exposures. The shaded band represents the modeled best fit (solid black line, r^2 ^= 0.82) using a four-parameter logistic equation [47] and the standard errors of the best-fitting coefficients to the model.

Once quantitative estimates of the light levels experienced by women at night have been determined, either for interpreting epidemiological studies or for designing parametric laboratory studies using animal models, the functional relationships between complex light exposures and melatonin suppression in humans must then be established. These relationships can then be meaningfully used to begin to assess the importance of LAN for suppressing nocturnal melatonin in actual architectural spaces. A model of human circadian phototransduction relating light exposure in laboratory environments to nocturnal melatonin suppression was developed by Rea and colleages [37]. Figure 3 is based upon that model and shows nocturnal melatonin suppression in humans as a function of the modeled circadian light stimulus. The solid line is the best fitting functional relationship between the modeled circadian light stimulus and nocturnal melatonin suppression values for humans from a number of empirical studies using both monochromatic and polychromatic light [35,41-45]. The circadian light stimulus values on the abscissa in Figure 3 are for a 60 minute light exposure, but, obviously, durations shorter or longer than 60 minutes will have less or more effect on melatonin suppression for the same circadian light level and spectrum [41]. In fact, the solid line in Figure 3 is based on data from 30 [43], 60 [35,41,44,45], and 90 minutes [42]; the data were adjusted empirically to account for those different durations. Also shown in this figure are the resulting circadian light stimulus values for varying illuminances of two conventional light sources, incandescent and daylight illuminants [46].

**Figure 4:**
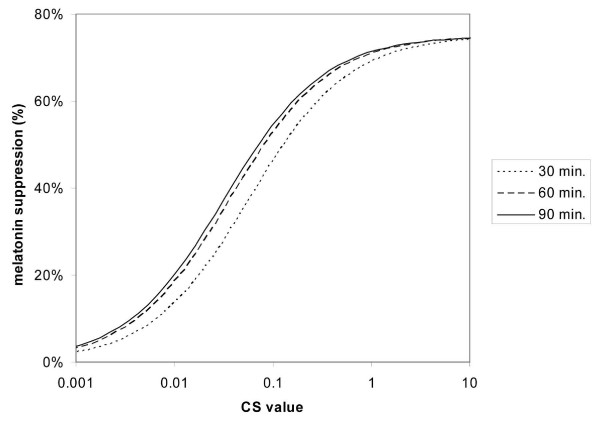
Predicted melatonin suppression as a function of circadian light stimulus (CS) [37] for different exposure durations, based on fitting the logistic-form curve in Figure 3 to the data separately for 30 minute [43], 60 minute [35, 41-45] or 90 minute [42] exposure durations.

Figure 4 shows the individual curves of the same form as in Figure 3, fitted to the 30-, 60- and 90-minute data separately. Table 2 shows the predicted values of nocturnal melatonin suppression for incandescent and daylight illumination, at different light levels and for different exposure durations; the values are based upon the three functions in Figure 4.

**Table 2:** Predicted Human Nocturnal Melatonin Suppression from Incandescent and Daylight Illumination [46] of Varying Corneal Illuminances and Durations, Based on Rea et al. [37]

*Incandescent*			
Illuminance (lx)	Melatonin suppression after 30 minutes	Melatonin suppression after 60 minutes	Melatonin suppression after 90 minutes

0.1	0%	0%	0%
0.3	0%	0%	0%
1	0%	1%	1%
3	1%	2%	2%
10	3%	5%	5%
30	8%	11%	13%
100	19%	25%	27%
300	35%	42%	45%
1000	54%	59%	60%
3000	65%	68%	69%

*Daylight*			

Illuminance (lx)	Melatonin suppression after 30 minutes	Melatonin suppression after 60 minutes	Melatonin suppression after 90 minutes

0.1	0%	0%	0%
0.3	0%	0%	1%
1	1%	1%	1%
3	2%	3%	4%
10	6%	9%	10%
30	14%	19%	20%
100	29%	36%	39%
300	47%	53%	55%
1000	62%	65%	66%
3000	69%	71%	71%

It should be noted that the 90-minute suppression values for the daylight source are consistent with those recently published by Zeitzer and colleagues [47], who found a half-maximal value of melatonin suppression in subjects exposed to three consecutive (over three days) 5-hour pulses of light at an illuminance (at the eyes) of around 100 lux. The uncertainty in the predicted values in Table 2 is, however, reflected in Figure 3. Empirically, for a specific circadian light stimulus value, the range in *mean *nocturnal melatonin suppression would be expected to be approximately 20%, or ± 10% of the predicted value. This level of uncertainty is also consistent with the confidence intervals for predicted melatonin suppression identified by Zeitzer and colleagues [47].

The studies of human nocturnal melatonin suppression to light in the laboratory illustrated in Figure 3 show that a reliable *threshold *for measurable melatonin suppression (approximately 15%, given the approximately ± 10% uncertainty in prediction of melatonin suppression) is, depending upon the spectral power distribution [37], about 30 lux of white light at the cornea for a 60-minute exposure [41,44,45]. Based then on the values in Table 2, it can be inferred that melatonin levels should be largely unaffected by brief (i.e., less than 30 minutes) exposures to the relatively low ambient levels of white (incandescent) light typically found in residences (i.e., less than 30 lux). Threshold values will undoubtedly be different for different individuals with different light histories [29-31] and with different demographic characteristics [48,49], but 30 lux for 30 minutes is proposed here as a *working expected threshold value *for women living in North American residences until more specific data are available. It is important to note again, however, that when additional task lights are used in the home for critical visual tasks, such as reading, sewing or grooming, light levels at the cornea *can *reach 150 to 200 lux at the eye [26]. Working under these light levels for several hours in the evening should in fact impact the onset of nocturnal melatonin production, but not necessarily in a clear or unambiguous way. Two studies have shown that LAN experienced by diurnally active adults in their homes has no impact on total nocturnal melatonin production, and only a small effect on phase [50,51]. On the other hand, in long sleepers (sleep duration longer than 9 hours) the duration of nocturnal melatonin production was greater than that in short sleepers (sleep duration less than 6 hours) under a constant routine protocol and exposure to less than 10 lux at the eye, although the amplitude of melatonin production was not significantly different [52]. Wehr [53] showed that the duration of nocturnal melatonin production was longer when subjects were exposed to a short photoperiod compared to a long photoperiod. Unfortunately, however, the quantity and spectrum of the light exposure was undefined, both indoors and outdoors. In sum, although it seems like long or short sleepers will differ in their nocturnal melatonin production duration, no studies have investigated whether this is due to individual differences (i.e., one is a long sleeper because he/she naturally produces melatonin for a longer duration over the course of the night) or if LAN typically found in real-life applications affects melatonin production duration in either long or short duration sleepers.

It is also important to stress that although a working threshold dose of light for nocturnal melatonin suppression in the home (i.e. 30 lux of white light for 30 minutes) could possibly be a useful starting point for discussion of a "safe" amount of light at night, it is clearly beyond our collective current understanding to suggest that this value, or any other, is a "safe" dose of light. It is equally premature to suggest that LAN is "unsafe," particularly without a proper, species-specific quantitative framework for such statements. What will be meaningful in the context of women's health is to base such discussions on existing data, or to generate new data, and to begin to move from a *qualitative *to a *quantitative *discussion of *circadian *LAN.

In the context of safety and health, it is also important to reconsider one purpose of LAN, namely to provide people with a "safe" nocturnal environment via properly specified emergency lighting [54,55] or security lighting [56,57]. Arguably it is safer to use light of modest intensity and duration to reduce risks of trips and falls during nocturnal visits to the bathroom and kitchen than to leave the lights off or to use lighting that is insufficient for visual navigation at home. In this context, however, safe navigation throughout the house does not require high light levels [55]. For example, vanity lights around a mirror operated by a switch located at the bathroom door usually provide much higher light levels at the eye than are necessary for safe navigation [26]. For safe movement through the house in the middle of the night it is usually only necessary to employ a secondary, low-level lighting system that does not exceed 30 lux of white light at the cornea for more than a few minutes. Some have suggested that only "red" dim night-lights be used, but this recommendation seems unnecessarily restrictive, especially because the visual system is not particularly sensitive to long-wavelength radiation and, therefore, safe navigation could be seriously hindered. In fact, utilizing light levels high enough for safe navigation but low enough to minimize circadian stimulation for short exposure times (less than 30 minutes) is especially important for senior adults who can have difficulty seeing and walking [58]. Falls among older adults have been associated with increased mortality [59]. To avoid risk of trips and falls due to temporary loss of sensitivity during dark adaptation after lights are shut off, a secondary, low-level lighting system for nighttime applications could, again, be quite important to health and well-being of senior adults [60].

It is also worth mentioning concerns expressed about the possible impact of light from the street or from adjacent properties entering the bedroom at night after lights in a residence have been turned off. These light levels rarely exceed 10 lux at the cornea *outdoors *(Table 1). Indoors, behind closed curtains, the levels would be likely to be much lower. Further, the human eyelids transmit only about 1% to 3% in the short wavelength region of the visible spectrum [61], so 1000 to 3000 lux at the closed eyelid would be needed for one hour to reach the threshold for melatonin suppression in humans. In fact, Jean-Louis and colleagues [62] showed that a one-hour exposure of 1700 lux of white light at the eyelids (delivered via a light mask) did not suppress nocturnal melatonin in humans. Hatonen and colleagues [63] showed that, on average, 2000 lux of white light applied to closed eyelids for 1 hour did not significantly suppress melatonin, although a quarter of their subjects did show some suppression. In this regard, individual differences may become a more important consideration in the future. Nevertheless, their conclusion was that indoor light was likely ineffective to the circadian system while one is asleep because light levels typically found in indoor environments are much lower than 2000 lux at the eye. Arguments for using black-out shades to limit light in the home at night because of its possible link to cancer risk have no quantitative foundation for melatonin suppression within the context of people living normal lives and working day-shifts. Of course, light trespass in our bedrooms should be avoided, but for the right reasons: light trespass is annoying and wasteful. Given the available published data on human melatonin suppression in response to light, light trespass through residential windows is an unlikely cause of melatonin suppression, simply because the light levels are so low, particularly with the eyes closed.

### Night-Shift Work as a Surrogate for LAN

Obviously, light is required to perform night-shift work. Because LAN can suppress nocturnal melatonin and because no instruments have previously been available to conveniently and accurately measure circadian light exposure, epidemiological studies have used night-shift work as one surrogate for LAN. In fact, studies on the relationship between LAN actually experienced by night-shift women and melatonin depletion have not been consistent with the assumption that LAN always suppresses melatonin [64,65]. Nevertheless, there should be a real societal concern about women performing night-shift work. As previously noted, a growing number of epidemiological studies show that night-shift work places women at a higher risk for breast cancer [9-11].

Measurements in several office and hospital workplaces (Table 1[27]) clearly show that vertical illuminances from white light likely to be experienced in the workplace for durations longer than 30 minutes can regularly exceed 30 lux. Measurements of light levels in neonatal intensive care units [66], for example, operated both day and night also showed that light levels could be high enough to result in nocturnal melatonin suppression (Table 2). It is reasonable to suppose then that night-shift women experience light levels bright enough and long enough to suppress nocturnal melatonin while they work at night. The data in Figure 2 suggest, if these women are typical, that the light levels to which they are exposed during the day are lower than those experienced at night, so that night-shift nurses do experience a regular, albeit muted and perhaps irregular, light-dark cycle. What is not yet clear, of course, is the circadian phase of these women with respect to their light-dark cycle. Data from Burgess and Eastman [50,51] suggest that the total melatonin levels across the 24-hour day might be unaffected by shortened sleeping schedules even though the phase of melatonin production is altered by both evening and morning light and by the sleep-wake cycle. In the context of night-shift work, little is known about the impact of long-term adaptation to different light-dark cycles over time [29,30]. LAN should not then be considered out of context of the light-dark cycle actually experienced by night-shift women and without a much better understanding of workers' adaptation to their working and sleeping schedule.

Night-shift women may also be at a higher risk of tumor growth for reasons apart from those associated with LAN. There are potential dietary differences between day- and night-shift workers [67] and this could be especially important if night-shift workers are consuming higher levels of linoleic acid [7]. Caffeine (100 mg) given four times a night was shown to reduce onset of melatonin levels in women in their luteal phase in the same manner as bright light [68]. Studies have also shown that sleep deprivation negatively impacts the immune system [69,70] and night-shift workers are generally sleep deprived because the quality of daytime sleep is often worse than nighttime sleep. It is important to add, however, that a recent epidemiological study failed to statistically show that occupational factors, such as sleep rhythms disruptions, were positively related to breast cancer risk among Finnish flight attendants [71]. Schernhammer and colleagues [72] also examined the relationship between melatonin and lifestyle factors and other endogenous sex steroid hormones. They concluded that age, body mass index, and heavy smoking were significantly related to lower levels of melatonin, while parity was significantly related to higher levels of melatonin. No other reproductive factors or sex steroid hormones were significantly associated with melatonin levels. Of course, more studies are needed to confirm their results. Moreover, although melatonin has been shown to have oncostatic effects (for review, see [73]), it may not be the only hormone important to breast cancer risk [74,75]. Clearly then, the higher risk of breast cancer associated with night-shift work is not related to LAN in a simple, easily defined way. Indeed, LAN associated with night-shift work may be entirely beneficial to night shift workers in helping entrain their circadian rhythms to the nighttime hours, and very different than LAN associated with diurnal lifestyles.

## Conclusion

Stevens [4] suspected that LAN in the built environment might be associated with increased incidence of breast cancer in industrialized societies. The present quantitative review and assessment of the circadian light stimulus experienced by diurnal women in their homes indicates that LAN, at least for those women, does not always approach the expected threshold for reliable melatonin suppression (Tables 1 and 2). Three points are important to consider, however. First, it is probably common for some diurnal women to experience light levels and durations above the expected threshold for nocturnal melatonin suppression, even in the home. However, the conditions and significance under which LAN above threshold might affect the duration and phase of melatonin production as well as the overall level of nocturnal melatonin production [50-53] are not well understood, especially in natural environments. Second, individual circadian light exposures over much longer periods than just 24 hours need to be considered in assessing a person's own sensitivity to light, its impact on melatonin suppression and, of course, risk of breast cancer. Third, our collective, current body of knowledge is entirely inadequate with regard to establishing "safe" or "unsafe" light levels and durations as they affect nocturnal melatonin suppression. Indeed, it is not known to what extent, if at all, circadian light exposure at night might impact breast cancer etiology in diurnal women.

The epidemiological evidence strongly suggests, however, that breast cancer is associated with night-shift work, but not necessarily with LAN exclusively [76]. Light during the solar night does regularly exceed the expected threshold for melatonin suppression in night-shift women, as would be expected, since they must perform their work at this time. During the solar day, when night-shift women would be expected to be sleeping, illuminance levels are generally lower than they are at night, but could possibly be influenced by daylight (e.g., Figure 2, top panel). In these night-shift women, lifestyle choices, including those during non-working days, almost certainly affect circadian phase and melatonin availability in ways uncharacteristic of diurnal women.

The link between melatonin suppression and cancer growth in laboratory animals nevertheless appears to be real and is undoubtedly important [20]. However, no studies have established a clear link between circadian light exposures commonly experienced by women at night and cancer etiology in rodents. Fundamentally, it will be impossible to understand the role of architectural LAN in breast cancer etiology until extended light exposure profiles experienced by women and girls in modern societies are properly characterized and then formally transformed into light exposure profiles for controlled studies of breast cancer using animal models. This has yet to be accomplished, but Bullough and colleagues [32] and Bierman and colleagues [28] have made significant steps toward making this bridge. Coupled with the pioneering studies of circadian regulation and breast cancer that have already been made [13,14,20,33] it may finally be possible to begin to determine what role architectural lighting might play in the growing incidence of breast cancer in industrialized societies.

## Abbreviations

aMT6s: 6-sulphatoxymelatonin

CI: confidence interval

DNA: deoxyribonucleic acid

LAN: light at night

## Competing interests

The author(s) declare that they have no competing interests.

## Authors' contributions

MGF and MSR drafted the main text. JDB drafted some sections of text. MGF and JDB developed the figures with substantial input from MSR.
